# BATF regulates collagen-induced arthritis by regulating T helper cell differentiation

**DOI:** 10.1186/s13075-018-1658-0

**Published:** 2018-08-02

**Authors:** Sang-Heon Park, Jinseol Rhee, Seul-Ki Kim, Jung-Ah Kang, Ji-Sun Kwak, Young-Ok Son, Wan-Su Choi, Sung-Gyoo Park, Jang-Soo Chun

**Affiliations:** 1 School of Life Sciences, Gwangju Institute of Science and Technology, Gwangju, 61005 Republic of Korea; 2 Keimyung University Dongsan Medical Center, Daegu, 41931 Republic of Korea

**Keywords:** BATF (basic leucine zipper transcription factor, ATF-like), Th cells, Collagen-induced arthritis, Fibroblast-like synoviocytes (FLS), Cartilage, Bone

## Abstract

**Background:**

We recently demonstrated that BATF, a member of the activator protein-1 (AP-1) family, regulates osteoarthritic cartilage destruction. Here, we explored the roles and regulatory mechanisms of BATF in collagen-induced arthritis (CIA) in mice.

**Methods:**

CIA and K/BxN serum transfer were used to generate inflammatory arthritis models in wild-type (WT) and *Batf*^*−/−*^ mice. RA manifestations were determined by examining CIA incidence, clinical score, synovitis, synovial hyperplasia, angiogenesis in inflamed synovium, pannus formation, bone erosion, and cartilage destruction. Immune features in RA were analyzed by examining immune cell populations and cytokine production.

**Results:**

BATF was upregulated in the synovial tissues of joints in which inflammatory arthritis had been caused by CIA or K/BxN serum transfer. The increases in CIA incidence, clinical score, and autoantibody production in CIA-induced WT mice were completely abrogated in the corresponding *Batf*^*−/−*^ DBA/1 J mice. Genetic ablation of *Batf* also inhibited CIA-induced synovitis, synovial hyperplasia, angiogenesis in synovial tissues, pannus formation, bone erosion, and cartilage destruction. *Batf* knockout inhibited the differentiation of T helper (Th)17 cells and the conversion of CD4^+^Foxp3^+^ cells to CD4^+^IL-17^+^ cells. However, BATF did not modulate the functions of fibroblast-like synoviocytes (FLS), including the expressions of chemokines, matrix-degrading enzymes, vascular endothelial growth factor, and receptor activator of NF-κB ligand (RANKL).

**Conclusion:**

Our findings indicate that BATF crucially mediates CIA by regulating Th cell differentiation without directly affecting the functions of FLS.

## Background

BATF (basic leucine zipper transcription factor, ATF-like) is a member of the activator protein-1 (AP-1) family whose members regulate various biological functions [1–3]. BATF, which lacks a transactivation domain, heterodimerizes with JUN to bind the AP-1 site for transcriptional regulation [3, 4]. We recently demonstrated that BATF regulates osteoarthritis (OA) in mice by modulating anabolic and catabolic gene expression in chondrocytes [4]. We found that overexpression of BATF upregulated matrix-degrading enzymes and downregulated cartilage matrix molecules; we also found that BATF expression in mouse joint tissues promoted OA cartilage destruction and that, conversely, knockout of *Batf* in mice (*Batf*^*−/−*^) suppressed experimental OA [4].

OA and rheumatoid arthritis (RA), which are the most common types of joint arthritis, share certain phenotypic features, such as cartilage destruction [5]. However, these diseases clearly differ in their etiologies, pathogenic mechanisms, and the cell types associated with each pathogenesis. OA is a degenerative joint disease that begins with the destruction of surface articular cartilage [6, 7]. Mechanical stresses (i.e., joint instability) and factors that predispose toward OA (i.e., aging) are important causes of OA pathogenesis [6, 7]. In contrast, RA is an inflammatory autoimmune disease that mainly targets the synovium, resulting in destruction of the joint architecture. Various cell types of joint tissues are associated with RA pathogenesis, including T cells, B cells, macrophages, synoviocytes, chondrocytes, and osteoclasts [8–10]. T cell-mediated autoimmune responses play a critical role in the RA pathogenesis, in which interleukin (IL)-17-producing T helper (Th) cells act as crucial effectors [8, 11, 12]. RA is characterized by synovitis with infiltration of immune cells, synovial hyperplasia that arises via proliferation of synovial cells, such as macrophage-like synoviocytes (MLS) and fibroblast-like synoviocytes (FLS), and angiogenesis in the hyperplastic synovium [8–10]. Synovial cells express numerous cytokines that have been implicated in many of the immune processes involved in RA [13]. RA manifestations also include erosion of the bone and cartilage which is caused by the formation of the pannus, which is an aggressive front of hyperplastic synovium. The pannus invades and destroys mineralized cartilage and bone through the action of osteoclasts [8–10].

BATF is known to regulate OA cartilage destruction, and we previously showed that BATF overexpression in joint tissues causes synovial inflammation [4] suggesting that BATF could contribute to inflammatory arthritis. This notion is supported by reports that proinflammatory cytokines, such as IL-1β and IL-6, increase BATF expression in naive CD4^+^ T cells [14, 15], BATF directly regulates IL-17 expression, and *Batf*-deficient mice show resistance to experimental autoimmune encephalomyelitis [16]. BATF also controls the development of follicular Th cells (Tfh) and class-switch recombination in B cells [17]. Additionally, inhibition of the transcriptional activity of c-Fos/AP-1 suppresses arthritic joint destruction in a mouse RA model [18]. However, while these previous reports suggest that BATF may be involved in RA pathogenesis, the role of BATF and its regulatory mechanisms are not yet well understood.

In this study, we examined whether BATF is required for collagen-induced arthritis (CIA), which is a commonly used experimental model of inflammatory arthritis caused by a T cell-dependent, antibody-mediated autoimmune response directed against cartilage type II collagen [19]. Here, we show that genetic ablation of *Batf* in mice suppresses the manifestations of CIA, including synovitis, synovial hyperplasia, angiogenesis in the inflamed synovium, and cartilage/bone erosion in the joint tissues. We also reveal that BATF regulates CIA by regulating Th cell differentiation without directly affecting the functions of FLS.

## Methods

### Mice and experimental RA

Male wild-type (WT) and *Batf*^*−/−*^ DBA/1 J mice were used to generate the CIA models. C57BL/6-background *Batf*^*−/−*^ mice [4] were backcrossed with DBA/1 J mice to generate *Batf*^*−/−*^ DBA/1 J mice. All mice were used in accordance with protocols approved by the Animal Care and Ethics Committees of the Gwangju Institute of Science and Technology. CIA was induced by a standard protocol [19, 20]. Mice were intradermally injected with incomplete Freund’s adjuvant alone (nonimmunized; NI) or Freund’s adjuvant containing 100 μg collagen type II (CIA). A booster injection was given 21 days later. The incidence and severity of arthritis were evaluated on the indicated days after the first immunization. Severity was evaluated using a clinical score (grade 0−4) of paw swelling [19, 20]. Joint tissues were fixed, decalcified with 0.5 M EDTA, embedded in paraffin, and sectioned at 5-μm thickness. Synovitis was evaluated by hematoxylin and eosin (H&E) staining, and synovial inflammation (grade 0−4) was scored as previously described [19, 20]. The pannus was visualized by H&E staining and quantified by scoring (grade 0−4) [19, 20]. Cartilage destruction was examined by safranin-O staining and scored using the OARSI (Osteoarthritis Research Society International) grading system [4, 20]. Inflammatory arthritis was also induced by K/BxN serum transfer [21] in WT and *Batf*^*−/−*^ C57BL/6 mice. Arthritic transgenic mice (K/BxN) and nontransgenic littermates (BxN) were generated by crossing KRN T cell receptor (TCR)-transgenic (K/B) mice with nonobese diabetic (NOD) mice. K/BxN and control sera were collected from K/BxN and BxN mice, respectively, and administered intraperitoneally to recipient mice on days 0 and 2. Mice were sacrificed on day 14 after serum transfer.

### Immunohistochemistry, immunofluorescence microscopy, and tartrate-resistant acid phosphatase (TRAP) staining

Antigens were retrieved by incubating joint sections at 60 °C overnight with sodium citrate buffer (10 mM sodium citrate, 0.05% Tween 20, pH 6.0). The sections were blocked with 2% bovine serum albumin in phosphate-buffered saline (PBS), and then incubated with primary antibodies, including rabbit anti-BATF (Brookwood Biomedical), rabbit anti-RANKL (receptor activator of NF-κB ligand) (Abcam), goat anti-IL-6 (R&D Systems), rabbit anti-TNF-α (tumor necrosis factor alpha) (Novus Biologicals), and rabbit anti-Ki67 (Abcam). The Dako REAL Envision Detection system was used for chromogenic color development. BATF-expressing cells in synovial tissues were identified by double immunofluorescence labeling of vimentin for FLS, CD11b for macrophages, CD4 for T cells, and B220 for B cells. The following primary antibodies were used: mouse anti-CD4, mouse anti-B220, rat anti-CD11b (Abcam), rabbit anti-BATF (ThermoFisher Scientific), and mouse anti-vimentin (BD Pharmingen). Blood vessels in synovial tissues were detected with mouse anti-CD31 (Dianova). TRAP activity was determined in joint sections as previously described [20, 22], and the numbers of TRAP-positive osteoclasts were counted in regions containing pannus-cartilage and pannus-bone interfaces.

### FLS culture and proliferation assays

FLS were isolated from WT or *Batf* knockout (KO) mice and cultured as described by Zhao et al. [23]. FLS of passages 4–8 were used for further analysis. Pure FLS (> 90% CD90^+^/< 1% CD14^+^) were identified by flow cytometry using antibodies against CD90 and CD14 (Abcam). FLS proliferation in culture was quantified by measuring bromodeoxyuridine (BrdU) incorporation [20]. Briefly, FLS cultured in a 96-well plate were treated with or without tumor necrosis factor (TNF)-α (100 ng/ml), and BrdU labeling was detected using the cell proliferation enzyme-linked immunosorbent assay (ELISA) BrdU kit (Roche). Proliferating cells in synovial sections were identified by detecting Ki67 using an antibody obtained from Abcam. The empty adenovirus (Ad-C) and BATF-expressing adenovirus (Ad-*Batf*) were as previously described [4]. FLS were infected with Ad-*Batf* and Ad-C at the indicated multiplicities of infection (MOI) for 2 h, washed, and maintained for 48 h before analysis.

### ELISA of autoantibody production

Collagen type II-specific antibodies were measured by ELISA [20]. Sera from NI and CIA mice were loaded to type II collagen-coated 96-well plates, incubated overnight at 4 °C, washed, and incubated for 1 h with alkaline phosphatase-labeled monoclonal antibodies against mouse IgG1, IgG2a, or IgG2b (Immunology Consultants Lab). *p-*Nitrophenyl phosphate was used as a substrate for chromogenic reactions, and the resulting color reaction was quantified using an ELISA plate reader.

### RT-PCR, qRT-PCR, and Western blotting

Total RNA was isolated from cultured FLS using the TRI reagent. The isolated RNA was reverse-transcribed, and the resulting cDNA was used for RT-PCR. The PCR primers and experimental conditions were as previously described for BATF, matrix metalloproteinase (MMP)3, and MMP13 [4], as well as CCL2, CCL5, CXCL1, CXCL5, CXCL10, GAPDH, RANKL, and vascular endothelial growth factor (VEGF) [20]. For Western blotting, FLS cells were incubated on ice for 30 min with radioimmune precipitation assay buffer (10 mM sodium phosphate, pH 7.2, 150 mM NaCl, 1% SDS, 1% deoxycholate, 1% Nonidet P-40). Whole-cell lysates were fractionated by polyacrylamide gel electrophoresis and immunoblotted using rabbit anti-BATF (Brookwood Biomedical) and goat anti-Lamin B (Santa Cruz).

### Flow cytometric analysis

Thymocytes, splenocytes, and lymphocytes were isolated from 6- to 8-week-old WT and *Batf*^*−/−*^ mice as previously described [20]. For detection of cell surface antigens, cells (1 × 10^6^) were labeled with fluorochrome-conjugated primary antibodies. For detection of intracellular antigens, surface-stained cells were fixed and permeabilized with a permeabilization buffer (eBioscience) or Foxp3/Transcription Factor Staining Buffer (eBioscience). The following antibodies were purchased from eBioscience for cell staining: Alexa Fluor 488®-conjugated anti-mouse CD4 and anti-mouse/rat Foxp3; FITC-conjugated anti-mouse TCRβ and anti-mouse CD8; PerCP Cy5.5-conjugated anti-mouse CD25, anti-mouse CD62L, and anti-mouse interferon (IFN)-γ; APC-conjugated anti-mouse CD4, anti-mouse B220, and anti-mouse IL-17A; PE-conjugated anti-human/mouse CD44, anti-mouse IL-4, anti-mouse CD25, and anti-mouse/rat Foxp3; and eFluor 450®-conjugated anti-mouse CD4, and PE-Cyanine7-conjugated anti-mouse CD4.

### In-vitro differentiation of Th cells

CD4^+^ T cell were isolated from the spleens of WT and *Batf*^*−/−*^ mice using an EasySep™ Mouse CD4^+^ T cell Isolation Kit (Stem Cell). The cells (2.5 × 10^5^) were cultured for 120 h under Th cell-differentiating conditions [23]. Briefly, for in-vitro differentiation of CD4^+^ T cells into Th1 cells, isolated cells were cultured with anti-mouse CD3 (5 μg/ml), anti-mouse CD28 (5 μg/ml), IL-12 (20 ng/ml), IL-2 (20 ng/ml), and anti-IL-4 (10 μg/ml). For in-vitro differentiation of CD4^+^ T cells into Th2 cells, isolated cells were cultured with anti-mouse CD3 (5 μg/ml), anti-mouse CD28 (5 μg/ml), IL-4 (25 ng/ml), IL-2 (20 ng/ml), anti-IFN-γ (10 μg/ml), and anti-IL-12R (10 μg/ml). For in-vitro differentiation of CD4^+^ T cells into Th17 cells, isolated cells were cultured with anti-mouse CD3 (5 μg/ml), anti-mouse CD28 (5 μg/ml), IL-6 (100 ng/ml), mouse-transforming growth factor (TGF)-β (5 ng/ml), anti-IFN-γ (10 μg/ml), and anti-IL-4 (10 μg/ml). For in-vitro differentiation of CD4^+^ T cells into Treg cells, isolated cells were cultured with anti-mouse CD3 (5 μg/ml), anti-mouse CD28 (5 μg/ml), mouse-TGF-β (5 ng/ml), and IL-2 (20 ng/ml). After the Th cell differentiation, cytokine production was analyzed by flow cytometry analysis after activation with phorbol-12-myristate 13-acetate (PMA; 50 ng/ml), ionomycin (500 ng/ml), and Brefeldin A solution (eBioscience) for an additional 4 h. To test the plasticity of Treg cells to Th17 cells in vitro, isolated cells were cultured with anti-mouse CD3 (5 μg/ml), anti-mouse CD28 (5 μg/ml), mouse-TGF-β (5 ng/ml), and IL-2 (20 ng/ml) for 2 days. Then, the cells were washed and further incubated with anti-mouse CD3 (5 μg/ml), anti-mouse CD28 (5 μg/ml), IL-6 (100 ng/ml), mouse-TGF-β (5 ng/ml), anti-IFN-γ (10 μg/ml), and anti-IL-4 (10 μg/ml) for 3 days.

### Cytokine analysis

For measurement of secreted cytokine levels, lymphocytes were isolated from the lymph nodes of CIA mice. Isolated lymphocytes (1 × 10^6^) were cultured for 4 h with PMA (50 ng/ml) and ionomycin (500 ng/ml) [24]. The LEGEND MAX mouse IL-6 ELISA kit and mouse IL-13 Platinum ELISA kit (Invitrogen) were used to detect IL-6 and IL-10, respectively. The BD Cytometric Bead Array solution (BD Biosciences) and a FACS Canto II flow cytometer (BD Biosciences) were used to measure secreted cytokines such as IL-2, IL-4, IL-10, IL-17A, IFN-γ, and TNF-α.

### Statistical analysis

The nonparametric Mann-Whitney *U* test was used for the analysis of data based on an ordinal grading system, such as the synovitis, pannus, and OARSI grades. For results obtained from ELISA and analyses of joint thickness, TRAP-positive cells, and BrdU incorporation, the data were first tested for conformation to a normal distribution using the Shapiro-Wilk test. The data were analyzed by the Student’s *t* test (pair-wise comparisons) or analysis of variance (ANOVA) with post-hoc tests (multi-comparisons) as appropriate. Significance was accepted at the 0.05 level of probability (*P* < 0.05).

## Results

### BATF is upregulated in synovial tissues of arthritic joints

**Fig. 1:**
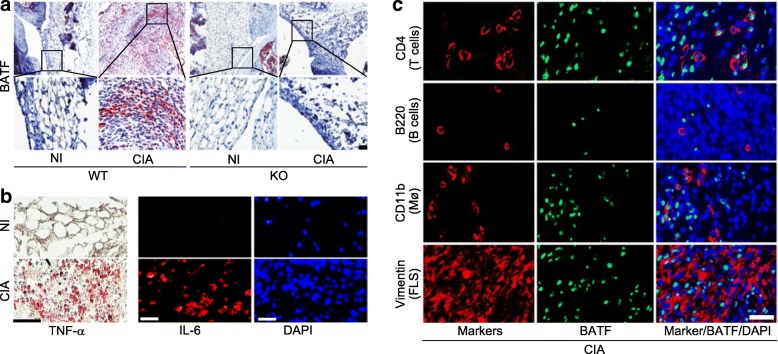
Upregulation of BATF in arthritic synovial tissues. **a** Representative images (*n* = 10) of BATF immunostaining in synovial tissue sections of nonimmunized (NI) and collagen-induced arthritis (CIA) wild-type (WT) DBA/1 J mice and *Batf*^*−/−*^ littermates (knockout (KO)). **b** Representative immunostaining images (*n* = 8) of tumor necrosis factor (TNF)-α and interleukin (IL)-6 in CIA synovial tissues determined by immunohistochemical staining and immunofluorescence microscopy, respectively. **c** Representative triple-immunofluorescence microscopic images of mouse CIA synovial sections (*n* = 12) immunostained for BATF, cell type-specific markers, and DAPI. Scale bars = 50 μm

To explore the possible functions of BATF in inflammatory arthritis, we first examined the expression patterns of BATF in the arthritic joints of DBA/1 J mice treated with CIA. Immunostaining revealed that BATF expression was markedly increased in the inflamed synovial tissues of CIA (Fig. 1a). In contrast, BATF was not detected (i.e., there was no obvious synovitis or synovial hyperplasia) in the synovial tissues of *Batf*^*−/−*^ mice subjected to CIA conditions (Fig. 1a). As expected, TNF-α and IL-6 were also markedly increased in the CIA synovial tissues (Fig. 1b). To identify cell types expressing BATF in synovial tissues, we performed double immunofluorescence staining of BATF with markers of various synovial cell types, including vimentin for FLS, CD4 for T cells, B220 for B cells, and CD11b for macrophages. BATF was detected in subsets of CD4^+^ T cells (> 80%), CD11b^+^ macrophages (~ 40%), and vimentin-expressing FLS (< 50%), but not in B220-expressing B cells (Fig. 1c).

### Genetic ablation of *Batf* inhibits CIA

**Fig. 2:**
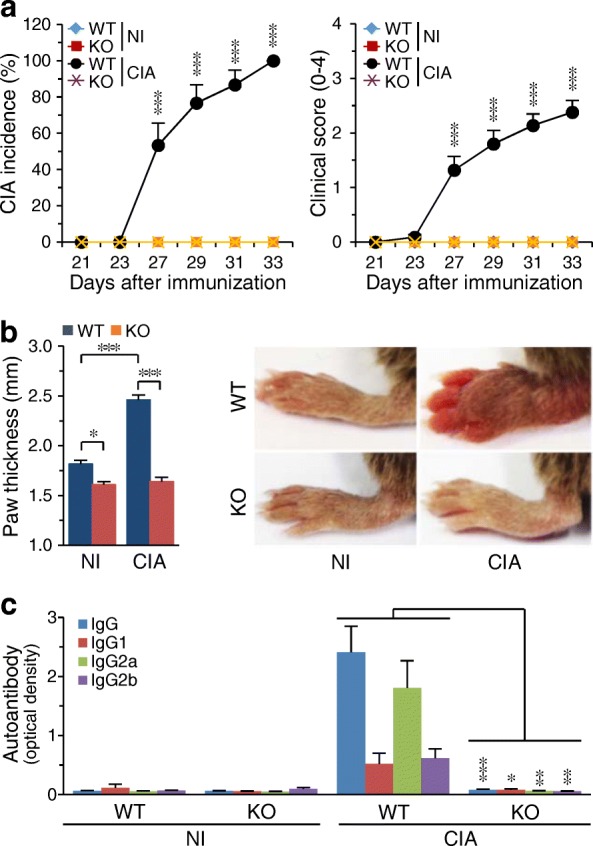
*Batf* KO inhibits CIA in DBA/1 J mice. **a** Incidence and severity of collagen-induced arthritis (CIA) symptoms in nonimmunized (NI) and CIA wild-type (WT) and *Batf*^*−/−*^ (knockout (KO)) DBA/1 J mice (*n* = 20 mice per group). **b** Typical paw images at 30 days after the first immunization, and paw thickness measured with a digital thickness caliper (*n* = 20 mice per group). **c** Type II collagen-specific autoantibody production in NI and CIA WT and *Batf*^*−/−*^ DBA/1 J mice (*n* = 10 mice per group). Values are means ± SEM; *P < 0.01, **P < 0.001, ***P < 0.0001

We next examined whether BATF regulates CIA. The CIA manifestations observed in WT mice, including CIA incidence, clinical scores, and paw swelling, were completely abrogated in *Batf*^*−/−*^ DBA/1 J littermates (Fig. 2a, b). The production of IgG autoantibodies against type II collagen is a key pathological change in RA pathogenesis, and the IgG2a autoantibody is particularly predominant in the CIA model [25]. Consistent with the above results, IgG2a production was markedly increased in the sera of CIA-induced WT mice, but this was completely suppressed in *Batf*^*−/−*^ littermates (Fig. 2c). These results collectively indicate that BATF is required for the pathogenesis of CIA in DBA/1 J mice.

### Genetic ablation of *Batf* inhibits CIA-induced synovitis, synovial hyperplasia, and angiogenesis in synovial tissues

**Fig. 3:**
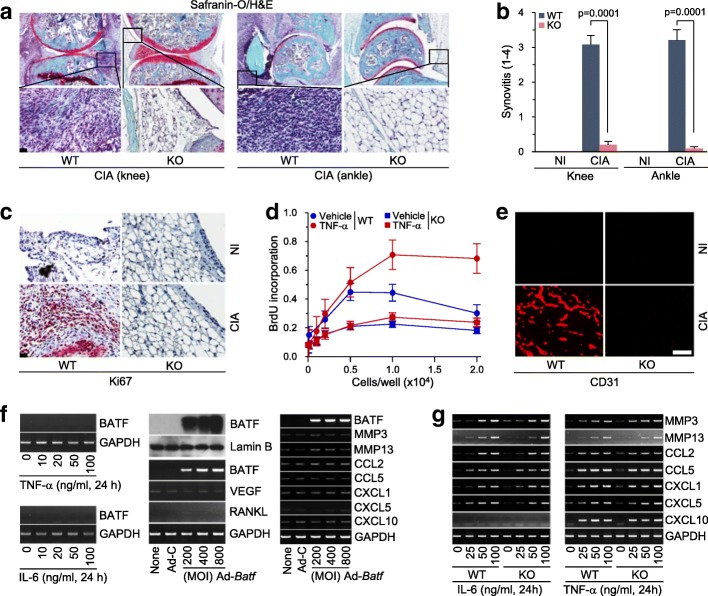
*Batf* KO inhibits CIA-induced synovitis, synovial hyperplasia, and angiogenesis. H&E staining (**a**) and scoring of synovial inflammation (*n* = 11) (**b**) in knee and ankle joints of nonimmunized (NI) and collagen-induced arthritis (CIA) wild-type (WT) and *Batf*^*−/−*^ (knockout (KO)) DBA/1 J mice. **c** Detection of cell proliferation by Ki67 staining in knee joint sections of NI and CIA WT and *Batf*^*−/−*^ DBA/1 J mice (*n* = 5). **d** Bromodeoxyuridine (BrdU) incorporation assays (*n* = 6) in FLS from WT and *Batf*^*−/−*^ DBA/1 J mice treated with vehicle or tumor necrosis factor (TNF)-α (100 ng/ml for 24 h). **e** Representative images (*n* = 6) of immunofluorescence microscopy of CD31, which was used to detect blood vessels in the ankle joints of NI and CIA WT and *Batf*^*−/−*^ DBA/1 J mice. **f** mRNA levels of the indicated molecules, as detected by RT-PCR in FLS isolated from NI synovium (*n* = 6). FLS were treated with or without the indicated concentrations of TNF-α or IL-6 (left), or with or without 400 MOI (multiplicity of infection) of empty adenovirus (Ad-C) or the indicated MOI of Ad-*Batf* (middle and right). BATF was also detected by Western blotting (middle). Lamin B was detected as a loading control. **g** WT and *Batf* KO FLS were treated with interleukin (IL)-6 or TNF-α. Indicated molecules were detected by RT-PCR (*n* > 5). Values are means ± SEM. Scale bars = 50 μm

CIA is characterized by a synovial hyperplasia that arises through the proliferation of synoviocytes, synovitis with infiltration of immune cells, and angiogenesis in synovial tissues [8–11]. Here, we found that CIA-induced synovitis was significantly blocked in *Batf*^*−/−*^ mice, as determined by H&E staining and inflammation scoring (Fig. 3a, b). To examine synovial hyperplasia, we determined synovial cell proliferation by Ki67 staining [26]. Ki67 was highly expressed in the synovial tissues of WT mice subjected to CIA-inducing conditions, whereas it was completely absent from those of the corresponding *Batf*^*−/−*^ mice (Fig. 3c). The inhibition of synovial cell proliferation in *Batf*^*−/−*^ mice was further confirmed by BrdU incorporation assays performed using dissociated FLS obtained from WT and *Batf*^*−/−*^ mice. Compared with WT FLS, *Batf*-deficient FLS showed significantly less proliferation in response to TNF-α (Fig. 3d).

Inflammatory synovial tissues express VEGF, which stimulates angiogenesis to maintain the chronic inflammatory status [27]. Consistent with the above results, neovascularization (as determined by CD31 staining) was observed in CIA-induced WT mice but not in *Batf*^*−/−*^ mice (Fig. 3e). Previous studies showed that FLS contribute to CIA by producing angiogenic factors associated with blood vessel formation [10, 20, 27, 28], and our group reported that hypoxia-inducible factor (HIF)-2α, which causes RA pathogenesis by modulating FLS functions, stimulates VEGF expression in FLS [20]. Here, we found that overexpression of BATF in FLS did not cause detectable expression of VEGF (Fig. 3f). Additionally, although BATF was upregulated in vimentin-expressing FLS of CIA synovium (Fig. 1c), treatment of FLS with IL-6 or TNF-α, cytokines that critically regulate RA pathogenesis [13], did not induce BATF expression (Fig. 3f). Furthermore, TNF-α- or IL-6-induced upregulation of matrix-degrading enzymes (MMP3 and MMP13), chemokines (CCL2 and CCL5), and chemokine receptors (CXCL1, CXCL5, and CXCL10) in FLS were not affected by *Batf* KO (Fig. 3g).

### Genetic ablation of *Batf* inhibits CIA-induced bone and cartilage erosion

**Fig. 4:**
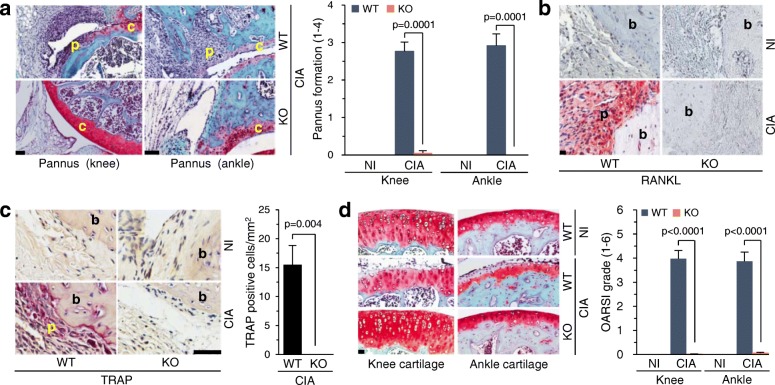
*Batf* KO inhibits CIA-induced bone erosion and cartilage destruction. **a** Representative images of pannus subjected to H&E and Safranin-O staining in NI and CIA WT and *Batf*^*−/−*^ DBA/1 J mice (left). Scoring of pannus formation (right; *n* = 9). **b** Representative images (*n* = 9) of receptor activator of NF-κB ligand (RANKL) immunostaining in ankle synovia of nonimmunized (NI) and collagen-induced arthritis (CIA) wild-type (WT) and *Batf*^*−/−*^ (knockout (KO)) DBA/1 J mice. **c** Tartase-resistant acid phosphatase (TRAP) staining and counting of TRAP-positive multinucleated cells (*n* = 6 mice per group) at the pannus-bone interface in ankle joints of NI and CIA WT and *Batf*^*−/−*^ DBA/1 J mice. **d** Cartilage destruction was detected by Safranin-O staining in NI and CIA WT and *Batf*^*−/−*^ DBA/1 J mice (left) and scored by OARSI grading (right; *n* = 12). Values are means ± SEM. Scale bars = 50 μm. b bone, c cartilage, p pannus

As CIA involves the formation of pannus, which invades and destroys mineralized cartilage and bone [8–11], we next examined the possible role of BATF in pannus formation and subsequent bone and cartilage destruction. Indeed, we found that the CIA-induced pannus formation found in WT mice was completely abrogated in *Batf*^*−/−*^ mice (Fig. 4a). Next, we examined the expression of RANKL, which promotes osteoclast differentiation to stimulate bone erosion [29, 30]. The induction of CIA in WT mice increased the expression of RANKL at the bone/pannus interface, but this was completely abrogated in *Batf*^*−/−*^ littermates (Fig. 4b). Although FLS are known to produce RANKL [20], BATF overexpression in FLS did not trigger any upregulation of RANKL (Fig. 3f), suggesting that the upregulation of BATF in the FLS of CIA synovium is not directly associated with RANKL expression. In addition, TRAP-positive osteoclasts, which were highly increased at the bone/pannus interface of WT mice, were not observed in *Batf*^*−/−*^ littermates (Fig. 4c). These results collectively suggest that *Batf* KO inhibits bone erosion under CIA-inducing conditions by blocking RANKL expression and osteoclast differentiation.

CIA in WT mice caused cartilage destruction, as determined by safranin-O staining and OARSI grading (Fig. 4d). Similar to the case of bone erosion, cartilage destruction was completely abrogated in *Batf*^*−/−*^ littermates (Fig. 4d). Cartilage destruction during CIA can be caused by pannus, which invades and destroys mineralized cartilage, or matrix-degrading enzymes produced by synoviocytes and chondrocytes [31, 32]. Although the matrix-degrading enzymes in inflammatory joint tissues are primarily produced by synoviocytes, such as FLS [31, 32], BATF overexpression in FLS did not cause any upregulation of the tested matrix-degrading enzymes (MMP3 and MMP13) or chemokines and chemokine receptors (CCL2, CCL5, CXCL1, CXCL5, and CXCL10) (Fig. 3f). Notably, however, we previously showed that BATF overexpression causes chondrocytes to produce MMP3 and MMP13 [4]. These results collectively suggest that matrix-degrading enzymes produced by chondrocytes, not FLS, may contribute to cartilage destruction during CIA.

### Genetic ablation of *Batf* does not affect thymic T cell development

**Fig. 5:**
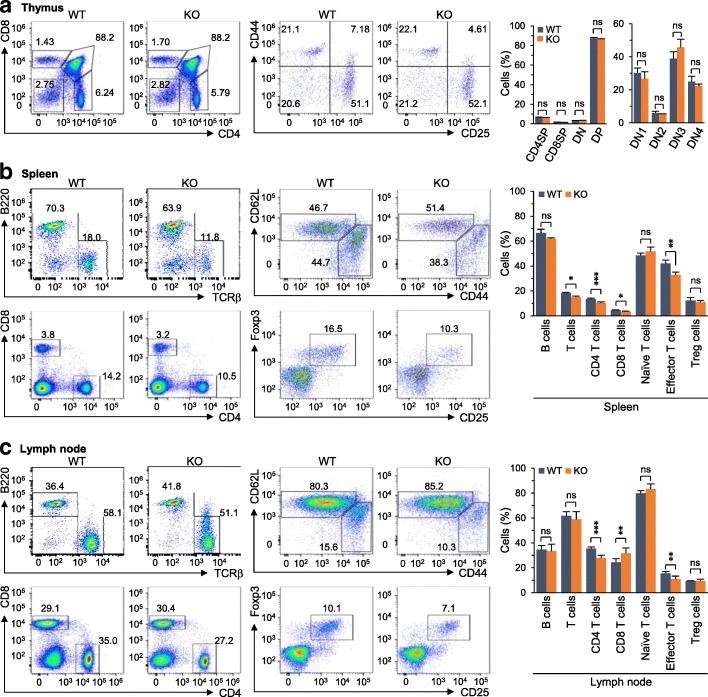
*Batf* KO does not affect thymic T cell development. **a**–**c** Representative flow cytometric analysis (*n* = 5 per group) and quantitation of immune cell populations. T cell development in thymus (**a**) and the populations of immune cells in the spleens (**b**) and lymph nodes (**c**) of wild-type (WT) and *Batf*^*−/−*^ (knockout (KO)) DBA/1 J mice were analyzed by flow cytometry. Values are presented as means ± SD; *P < 0.01, **P < 0.001, ***P < 0.0001. ns not significant

Given that T cells, such as Th17 cells, play crucial roles in inflammatory arthritis [33], we examined whether *Batf* deficiency affected T cell development in DBA/1 J mice. Flow cytometric analysis revealed that thymic T cell development was not affected by *Batf* deletion in DBA/1 J mice (Fig. 5a). However, consistent with a previous report [34], *Batf*^*−/−*^ mice exhibited altered T cell populations in peripheral organs, such as the spleen and lymph nodes (Fig. 5b, c). Compared with WT mice, T cells were slightly reduced in the spleen (Fig. 5b) and lymph nodes (Fig. 5c) of *Batf*^*−/−*^ mice. CD4^+^ effector/memory T cells were also slightly reduced in the spleen (Fig. 5b) and lymph nodes (Fig. 5c) of *Batf*^*−/−*^ mice. Overall, although the populations of T cell subsets in the periphery were altered by *Batf* deletion, the degree of alteration was small (i.e., less than 10%) (Fig. 5b, c).

### *Batf* KO modulates Th cell differentiation

**Fig. 6:**
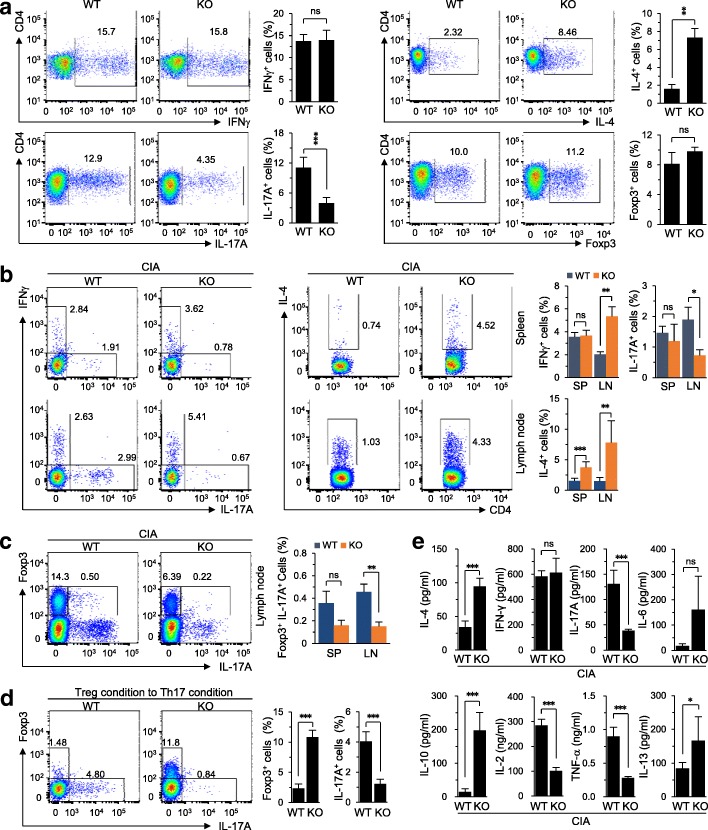
*Batf* KO reduces Th17 differentiation in CIA. **a** Populations of Th1, Th2, Th17, and Treg cells differentiated from uncommitted CD4^+^ T cells of wild-type (WT) and *Batf*^*−/−*^ (knockout (KO)) DBA/1 J mice (*n* = 5). **b** Analysis of interferon (IFN)-γ, interleukin (IL)-17A, and IL-4 producing CD4^+^ T cells in spleens (SP; top panel) and lymph nodes (LN; bottom panel) of WT and *Batf*^*−/−*^ DBA/1 J mice 4 to 5 weeks later from CIA induction (*n =* 5 mice per group). **c** Flow cytometric analysis of the productions of Foxp3 and IL-17A in CD4^+^ T cells from lymph nodes of WT and *Batf*^*−/−*^ DBA/1 J mice 4 to 5 weeks later from CIA induction (*n =* 5 mice per group) under CIA conditions. **d** Populations of Foxp3^+^ and IL-17A^+^ cells in CD4^+^ T cells cultured under Treg differentiation condition to Th17 differentiation condition (*n* = 5). **e** Cytokine production of total lymph node cells from WT and *Batf*^*−/−*^ DBA/1 J mice 4 to 5 weeks later from CIA induction (*n* ≥ 5 mice per group). Values are presented as means ± SD; *P < 0.01, **P < 0.001, ***P < 0.0001. ns not significant

To further elucidate the functions of BATF, we examined whether *Batf* deficiency affects the in-vitro differentiation of CD4^+^ T cells into Th cells, including Th1 (CD4^+^IFNγ^+^), Th2 (CD4^+^IL-4^+^), Th17 (CD4^+^IL-17A^+^), and Treg cells (CD4^+^Foxp3^+^). Interestingly, *Batf* deletion significantly increased Th2 cell differentiation in *Batf*^−/−^ mice, while in-vitro Th1 and Treg cell differentiations were not affected by *Batf* gene deletion (Fig. 6a). *Batf* gene deletion also significantly reduced Th17 cell differentiation in *Batf*^−/−^ DBA/1 J mice compared with WT control mice (Fig. 6a). Consistent with this finding, the Th17 cell population was significantly reduced in the lymph nodes of *Batf*^*−/−*^ mice subjected to CIA-inducing conditions (Fig. 6b). Moreover, the CD4^+^Foxp3^+^IL-17^+^ cell population was dramatically reduced in the peripheral lymph nodes of CIA-induced *Batf*^−/−^ mice (Fig. 6c). As the transconversion of Treg cells to Th17 cells can reportedly exacerbate Th17-mediated inflammation [35], we examined whether BATF affects the conversion of Treg cells to Th17 cells in vitro. Indeed, BATF deficiency dramatically abrogated the transconversion of Treg cells to Th17 cells (Fig. 6d), indicating that BATF regulates this conversion. Finally, we analyzed cytokine production in the lymph nodes of *Batf*^*−/−*^ mice and WT littermates under CIA-inducing conditions. Consistent with the increase in Th2 cell differentiation, the production of the Th2 cytokines IL-4, IL-10, and IL-13 were increased in CIA-induced *Batf*^*−/−*^ mice compared with WT littermates (Fig. 6e). Additionally, IL-6 was also increased in CIA-induced *Batf*^*−/−*^ mice (Fig. 6e). In contrast, the production of the inflammatory cytokines IL-2, IL-17, and TNF-α were reduced in *Batf*^*−/−*^ mice under CIA-inducing conditions (Fig. 6e).

### Genetic ablation of *Batf* does not affect inflammatory arthritis caused by K/BxN serum transfer

**Fig. 7:**
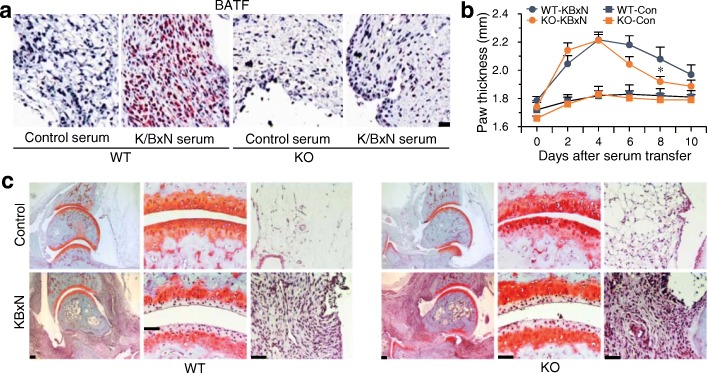
*Batf* KO does not affect inflammatory arthritis caused by K/BxN serum transfer. **a** Representative immunostaining images (*n* = 6) of BATF immunostaining in synovial tissue sections of C57BL/6 mice transferred with control or K/BxN serum. **b** Paw thickness measured with a digital thickness caliper in wild-type (WT) or *Batf* knockout (KO) mice transferred with control (Con) or K/BxN serum (*n* = 12 mice per group). **c** Typical images of cartilage destruction and synovitis detected by Safranin-O and H&E staining in WT or *Batf* KO mice transferred with control or K/BxN serum (*n* = 12 mice per group). Values are means ± SEM. Scale bars = 50 μm

Finally, we examined a possible role of BATF in T cell-independent inflammatory arthritis using C57BL/6 mice subjected to K/BxN serum transfer [21]. Immunostaining revealed that BATF expression was markedly increased in the inflamed synovial tissues caused by K/BxN serum transfer (Fig. 7a). However, all examined manifestations of inflammatory arthritis caused by K/BxN serum transfer in WT mice (i.e., paw thickness, synovial inflammation, and cartilage erosion) were not markedly inhibited in *Batf* KO mice (Fig. 7b, c). Our results suggest that BATF is not essential for T cell-independent inflammatory arthritis.

## Discussion

We herein show that BATF is required for CIA since *Batf*^*−/−*^ mice exhibit complete suppression of the manifestations of CIA. We further demonstrate that BATF regulates Th cell differentiation during CIA. The pathogenesis of RA is associated with enrichment of Th17 cells in the inflamed synovium and enhancement of IL-17 production [8, 9, 36, 37]. A recent report indicated that the conversion of Treg cells to Th17 cells contributes to bone destruction in CIA mice [35], suggesting that an imbalance between these cell populations contributes to CIA. BATF is known to regulate Th17 cell differentiation by modulating the expression of the transcription factor RORγt [16], and to control class-switch recombination in T and B cells [17]. Thus, BATF was previously known to function at multiple hierarchical levels in two cell types to globally regulate switched-antibody responses. Here, we demonstrated that BATF deficiency decreases Th17 cell differentiation both in vivo and in vitro. It was previously reported that a portion of Foxp3-positive cells also expresses IL-17, and that CD4^+^Foxp3^+^IL-17^+^ cells can be found in CIA mice [35]. However, we found here that *Batf* deficiency completely abolished CD4^+^Foxp3^+^IL-17^+^ cell generation under CIA-inducing conditions. An additional interesting finding of our current study is that BATF regulates Th2 cell differentiation; we demonstrated that IL-4-producing CD4^+^ T cells were increased in CIA-induced *Batf*^*−/−*^ mice compared with WT control mice. This is consistent with a previous report that IL-4 negatively regulates the induction and progression of CIA [38]. In addition, BATF is not detected in the infiltrated B cells of CIA synovial tissue. Although this does not rule out expression and the possible role of BATF in B cells of peripheral lymphatic tissues, our results indicate that upregulation of BATF in the infiltrated B cells is not essential for pathogenesis of CIA. Furthermore, we found that T cell-independent inflammatory arthritis caused by K/BxN serum transfer was not affected by *Batf*^*−/−*^ mice. Based on these findings, we propose that BATF deficiency alters Th cell differentiation, enabling *Batf*^*−/−*^ mice to resist CIA. Thus, BATF inhibition could be a useful strategy for the treatment of RA.

We also report that FLS are not directly associated with the BATF-mediated regulation of CIA, although BATF appears to regulate the proliferation of FLS in response to TNF-α. Accumulating evidence indicates that FLS are key players in RA pathogenesis [10, 28]. Cytokines and chemokines produced by FLS attract T cells to RA synovium, and the interaction of FLS with T cells results in the activation of both cell types. FLS also produce matrix-degrading enzymes involved in cartilage destruction, angiogenic factors associated with neovascularization, and RANKL [10, 28]. The latter factor regulates osteoclastogenesis, which requires physical contact of precursor cells with RANKL-expressing FLS or T cells [29, 30, 39]. We showed previously that HIF-2α modulates various functions of FLS, including proliferation, the expressions of RANKL and various catabolic factors, and osteoclastogenic potential [20]. Here, we found that, unlike HIF-2α, TNF-α and IL-6 do not cause BATF expression in FLS, and BATF overexpression in FLS does not modulate the expressions of various matrix-degrading enzymes or chemokines. These results collectively support our notion that the ability of BATF to regulate CIA is not due to a direct BATF-mediated modulation of FLS functions.

It has proven difficult to elucidate the role of chondrocytes in cartilage destruction during RA pathogenesis [5]. Such destruction occurs primarily at the interface of the pannus and calcified cartilage [5, 40]. There is evidence that proteoglycans are lost from the superficial zone, where cartilage contacts with synovial fluid, but not with the pannus [5]. Because RA synovium produces various matrix-degrading enzymes [5, 8, 9], cartilage destruction at the superficial zone may be due to synovial cell functions. However, proteoglycans can also be lost from the middle and deep zones of the cartilage [5], suggesting that a chondrocyte may help degrade its own matrix by releasing matrix-degrading enzymes. Indeed, we recently demonstrated that BATF upregulates MMP3 and MMP13 in chondrocytes, leading to cartilage destruction during OA pathogenesis [4]. Therefore, our current and previous [4] results suggest that the BATF-mediated regulation of MMP3 and MMP13 expression in chondrocytes is associated with cartilage destruction during CIA.

The results of the present study collectively indicate that BATF regulates CIA in mice, and our previous work showed that BATF functions as a catabolic regulator of OA cartilage destruction by upregulating catabolic enzymes (e.g., MMP3 and MMP13) in chondrocytes [4]. Thus, despite their different etiologies and pathogeneses, both RA and OA are regulated by BATF. However, different mechanisms are involved; BATF regulates OA pathogenesis by upregulating matrix-degrading enzymes in chondrocytes, whereas it appears to regulate RA pathogenesis by regulating Th cell differentiation. Thus, BATF could be a useful target for the regulation of RA.

## Conclusions

In summary, we demonstrated here that BATF regulates CIA, including synovitis, synovial hyperplasia, angiogenesis in the inflamed synovium, cartilage destruction, and bone erosion in the joint tissues. We also reveal that BATF regulates CIA by regulating Th cell differentiation without directly affecting the functions of FLS.

## Abbreviations

AP-1: Activator protein-1
BATF: Basic leucine zipper transcription factor, ATF-like
BrdU: Bromodeoxyuridine
CIA: Collagen-induced arthritis
ELISA: Enzyme-linked immunosorbent assay
FLS: Fibroblast-like synoviocytes
H&E: Hematoxylin and eosin
HIF: Hypoxia-inducible factor
IFN: Interferon
IL: Interleukin
KO: Knockout
MLS: Macrophage-like synoviocytes
MMP: Matrix metalloproteinase
NI: Nonimmunized
OA: Osteoarthritis
PMA: Phorbol-12-myristate 13-acetate
RA: Rheumatoid arthritis
RANKL: Receptor activator of NF-κB ligand
TCR: T cell receptor
TGF: Transforming growth factor
Th: T helper
TNF: Tumor necrosis factor
TRAP: Tartase-resistant acid phosphatase
VEGF: Vascular endothelial growth factor
WT: Wild-type
